# A Review of Aeruginascin and Potential Entourage Effect in Hallucinogenic Mushrooms

**DOI:** 10.1192/j.eurpsy.2022.2297

**Published:** 2022-09-01

**Authors:** P. Chue, A. Andreiev, E. Bucuci, C. Els, J. Chue

**Affiliations:** 1 UNIVERSITY OF ALBERTA, Psychiatry, EDMONTON, Canada; 2 UNIVERSITY OF OTTAWA, Psychiatry, OTTAWA, Canada; 3 UNIVERSITY OF ALBERTA, Clinical Trials And Research Program, EDMONTON, Canada

**Keywords:** ENTOURAGE EFFECT, Psilocybin, Aeruginascin

## Abstract

**Introduction:**

The 5-HT_2A_ agonist classic psychedelic, psilocybin (O-phosphoryl-4-hydroxy-N,N-dimethyltryptamine) is a tryptophan, indole-based alkaloid present in up to 2% of certain hallucinogenic “magic” mushroom species; typically Psilocybe azurescens, semilanceata, and cyanescens,. In addition, mushrooms may contain psilocin (4-hydroxy-N,N-dimethyltryptamine). Both are indolylalkylamines (tryptamines); other naturally occurring tryptamine compounds include norbaeocystin, baeocystin, norpsilocin, and aeruginascin. A putative synergistic contribution of these compounds has been referred to as the “entourage” effect. Aeruginascin (N,N,N-trimethyl-4-phosphoryloxytryptamine) is found naturally in Inocybe aeruginascens and Pholiotina cyanopus mushroom species and ingestion reportedly invokes elevation in mood without accompanying hallucinogenic effects:

**Objectives:**

To review the pharmacology of aeruginascin and putative entourage effect.

**Methods:**

The extant literature on aeruginascin was reviewed and discussed.

**Results:**

Methylation of aeruginascin results in an active metabolite, 4-hydroxy-N,N,N-trimethyltryptamine (4-HO-TMT) which has been shown to bind at 5-HT_1A_, 5-HT_2A_, and 5-HT_2B_ receptors with Inhibition Constants (K_i_) of 4400, 670, and 120 nM respectively; compared with psilocybin’s binding of 567.4, 107.2 and 4.6 nM respectively. Further, 4-HO-TMT does not bind at the 5-HT_3_ receptor, and as a quaternary trimethylammonium compound it is less likely to be able to cross the blood-brain-barrier (BBB).

**Conclusions:**

There are very limited data with respect to the pharmacology of aeruginascin. Its activity at serotonin receptors is less by several orders of magnitude than psilocybin and it has potentially less brain penetrance. Given that it is found in different mushrooms species the data would suggest that its direct contribution to any entourage effect is limited. Further research in needed into other naturally occurring tryptamine compounds.

**Disclosure:**

PC is a member of the Scientific Advisory Board of Zylorion. AA, EB, JC, CE have no disclosures to report.

